# Effect of Carbon Fillers on the Surface Resistivity and Mechanical Properties of Silicone Materials

**DOI:** 10.3390/ma19214537

**Published:** 2026-10-28

**Authors:** Ewelina Chmielnicka, Małgorzata Szymiczek, Weronika Prusak

**Affiliations:** 1 Faculty of Mechanical Engineering, Silesian University of Technology, 18A Konarskiego Street, 44-100 Gliwice, Poland; malgorzata.szymiczek@polsl.pl; 2 Faculty of Chemical Engineering and Technology, Tadeusz Kościuszko Cracow University of Technology, 24 Warszawska Street, 31-155 Kraków, Poland; weronika.prusak@student.pk.edu.pl

**Keywords:** poly(dimethylsiloxane) (PDMS), conductive carbon black, electrical percolation

## Abstract

This study investigated the influence of carbon fillers with different morphologies on the physicomechanical and electrical properties of composites based on polyaddition-cured poly(dimethylsiloxane) (PDMS). Conductive carbon black, flake graphite, and reduced graphene oxide (rGO) were introduced individually into the silicone matrix. In addition, selected multicomponent formulations containing conductive carbon black, flake graphite, and a viscosity modifier were prepared. The materials were characterized in terms of density, Shore A hardness, tensile properties, surface resistivity, microstructure, and thermal properties using differential scanning calorimetry (DSC). Conductive carbon black had the most pronounced effect on surface resistivity. At a filler content of 2.5 wt.%, the composite retained its insulating properties, whereas increasing the carbon black content to 5 wt.% reduced the mean surface resistivity to approximately 10^6^ Ω/sq, suggesting that the electrical percolation threshold likely lies between these concentrations. Within the investigated concentration ranges, flake graphite and rGO did not lead to the formation of effective conductive pathways. The incorporation of carbon fillers generally resulted in a reduction in tensile strength and elongation at break, with the most pronounced deterioration in mechanical properties observed for composites containing conductive carbon black. In contrast, flake graphite caused a clear increase in hardness with increasing filler content. DSC analysis showed that conductive carbon black had a strong effect on the enthalpy of the observed thermal transition, while only slightly affecting its characteristic temperature. The results confirm that the electrical and mechanical properties of PDMS composites are strongly determined by the morphology and content of carbon fillers, as well as by their mutual interactions.

## 1. Introduction

Organosilicon materials constitute an important group of engineering materials used in applications requiring elasticity, electrical insulation, and thermal and chemical stability. This group includes silicone materials based on poly(dimethylsiloxane) (PDMS). Their characteristic chemical structure consists of a siloxane Si–O–Si backbone [1] with organic side groups. This type of structure provides high conformational freedom of the backbone, which facilitates the mobility of polymer chains [2,3] and underlies the flexibility of PDMS over a wide range of environmental conditions.

PDMS-based silicones are attractive materials owing to their favorable properties, including thermal and chemical resistance [4,5]; however, their inherent characteristics do not always meet the increasing demands of industry and more advanced applications [6]. Consequently, numerous studies are currently focused on modifying PDMS silicones to tailor their properties for applications such as electronic components [7], sensors [8], and medical devices [9].

One of the characteristic properties of unmodified PDMS-based silicones is their high electrical resistivity, which makes them excellent electrical insulators. Current research therefore focuses on introducing selected electrically conductive fillers into the polymer matrix [10,11]. Such modification of the PDMS matrix may enable the development of materials combining flexibility, chemical and environmental resistance, and electrical conductivity [12]. Tailoring PDMS toward electrically conductive behavior may significantly expand its applicability in demanding engineering applications. Research conducted in recent years indicates that one of the most promising approaches involves the use of carbon-based fillers, as they enable substantial modification of electrical properties while maintaining relatively low density [13] and, to some extent, the ability to undergo large deformations [14].

Changes in the electrical properties of polymer composites containing conductive particles are directly related to the structure formed by the filler within the polymer matrix. At low filler concentrations, individual particles or aggregates remain separated from one another by layers of the insulating silicone polymer, and the material therefore retains high electrical resistivity. As the filler concentration increases, the average distance between the particles decreases, increasing the probability of direct particle-to-particle contact and charge transport between neighboring conductive regions. Once a critical filler concentration is reached, a continuous three-dimensional conductive network may be formed, which can be accompanied by a sharp increase in electrical conductivity [15,16].

One of the most widely used carbon-based conductive fillers is conductive carbon black (CB). Its major advantages include broad industrial availability and relatively low cost. The incorporation of carbon black into PDMS can affect not only the electrical properties but also the physicomechanical behavior of the material [17,18]. Another relatively readily available carbon material is flake graphite. In contrast to carbon black, which consists of small primary particles forming aggregates, graphite exhibits a layered structure and platelet-like morphology. Such geometry can substantially affect the formation of conductive pathways. Contact between the surfaces of adjacent graphite platelets may promote the development of two- and three-dimensional conductive networks; however, the efficiency of this process depends, among other factors, on the degree of filler dispersion, platelet orientation, and the extent of interparticle contact [19,20,21,22]. Graphene-based materials, including reduced graphene oxide (rGO), are characterized by a two-dimensional geometry, a high aspect ratio, and a large specific surface area. These features mean that even relatively small amounts of this type of filler may significantly affect the structure of the composite [23,24,25,26].

Filler dispersion therefore remains an important issue in the design of electrically conductive PDMS composites. Even a material exhibiting very high intrinsic electrical conductivity does not necessarily provide high conductivity of the resulting composite if it forms isolated agglomerates rather than a continuous conductive network. From the perspective of practical applications, a key challenge is to achieve the required electrical properties while retaining the mechanical characteristics typical of silicone materials. Increasing the filler content generally facilitates the development of a conductive network; however, it may simultaneously increase stiffness and hardness and reduce elongation at break [27,28,29].

A comparison of carbon fillers with different structures and morphologies within the same silicone matrix and under comparable sample preparation conditions may reveal differences that determine both their ability to form conductive pathways and their influence on the restriction of polymer-chain mobility [30,31,32,33].

The aim of this study was to determine the effect of the type and content of carbon fillers conductive carbon black, flake graphite, and reduced graphene oxide (rGO) on the electrical and mechanical properties of PDMS-based materials. Particular attention was given to surface resistivity as a parameter characterizing the ability to develop a conductive structure within the material. This approach enables evaluation of the relationships between filler type and content, the development of an electrically conductive network within the composite, and the retention of the characteristic elastic properties of PDMS.

## 2. Materials and Methods

The experiments used addition-cured room temperature-vulcanizing (RTV) poly(dimethylsiloxane) 3428 (designation: AA), supplied by Elkem (Oslo, Norway).

The material properties are summarized in Table 1.

A commercial silicone formulation was used to minimize the influence of additional modifiers. The silicone was not chemically modified in any way; the investigated fillers and, where indicated, a viscosity modifier were incorporated into the matrix. The selected silicone belongs to the group of addition-cured silicones, in which crosslinking occurs through a reaction between two base components, A and B. Combining these components results in the formation of a permanent crosslinked network.

The following carbon-based fillers and viscosity modifier were used to prepare the PDMS composites:

- MG 394 flake graphite, characterized by a layered crystalline structure and a particle (flake) size below 45 µm. The graphite particles exhibit a platelet-like morphology with a high lateral dimension-to-thickness ratio. The thermal conductivity of graphite ranges from 140 to 233 W/(m·K).
- PRINTEX^®^ XE2-B conductive carbon black, supplied by Orion Engineered Carbons (Spring, TX, USA), characterized by approximately spherical primary particles with a mean diameter of approximately 30 nm. The material exhibits a highly developed aggregate structure and a large BET specific surface area of approximately 1000–1100 m^2^/g.
- Reduced graphene oxide (rGO), supplied as a black powder with a purity of 99%, characterized by flake sizes ranging from 1 to 5 µm.
- BLUESIL FLD 47V50 (Elkem Silicones) is a clear, non-reactive silicone fluid with a viscosity of approximately 50 mPa·s. Among its applications, this product is used to reduce the viscosity of two-component RTV silicone systems. In the present study, it was used to improve the flowability of filled PDMS mixtures.

The test materials were prepared under laboratory conditions in accordance with the manufacturer’s recommendations regarding the proportions of the silicone base-to-catalyst ratio (Table 1). The materials were mixed using a High-Speed Dissolver Dispermat LC30 (VMA-Getzmann GmbH, Reichshof, Germany) at a mixing speed of 1500 rpm for 5–15 min. The intensive homogenization process resulted in considerable air entrapment within the materials. To minimize the potential adverse effects of voids within the specimen cross-sections, the mixtures were vacuum-degassed at 0.06 MPa for 5 min prior to casting. The degassed mixtures were then cast into polyethylene molds measuring 100 × 100 mm, with thicknesses of 4 mm and 2 mm.

The prepared materials were conditioned at 23 ± 5 °C for 24 h, after which test specimens were cut in accordance with the requirements of the relevant standards.

Table 2 lists the sample designations and compositions.

The fillers used in the investigated composites were selected to enable an assessment of the effects of filler type, filler content, and combinations of carbon-based fillers on the physicomechanical properties of the material. Particular attention was given to the possibility of forming a continuous electrically conductive network. Conductive carbon black, flake graphite, and reduced graphene oxide (rGO) were used as potentially conductive phases. These fillers differ in particle size, morphology, and specific surface area.

The unfilled PDMS served as the reference material to determine the baseline electrical properties of the polymer matrix. In addition, a sample containing 10% of the ZL viscosity modifier was prepared to provide a reference for assessing the effect of the modifier alone. The introduction of the viscosity modifier was also important from the processing point of view, particularly for highly filled materials, because the increased flowability of the mixtures may facilitate filler incorporation and mixing.

The multicomponent composites containing both flake graphite and conductive carbon black were prepared to explore the combined effects between fillers differing in morphology and particle size. In such a system, the larger graphite flakes could contribute to the framework of a conductive network, while the much smaller carbon black aggregates may occupy the spaces between them and create additional electrical connections between neighbouring graphite particles.

The selection of conductive carbon black contents in the multicomponent formulations was based on the concentration range in which a sharp decrease in surface resistivity was observed, suggesting the occurrence of an electrical percolation threshold. The incorporation of flake graphite at concentrations of 10 and 20 wt.% enabled a comparison of formulations containing different amounts of a filler with a distinct morphology and an evaluation of its combined effect with conductive carbon black.

The sample set thus allowed the effects of individual carbon fillers to be compared with those of selected multicomponent formulations. The use of fillers with different geometries and dimensional scales was intended to increase the probability of forming an effective three-dimensional conductive network while maintaining the carbon phase content at a reasonably low level.

**Figure 1:**
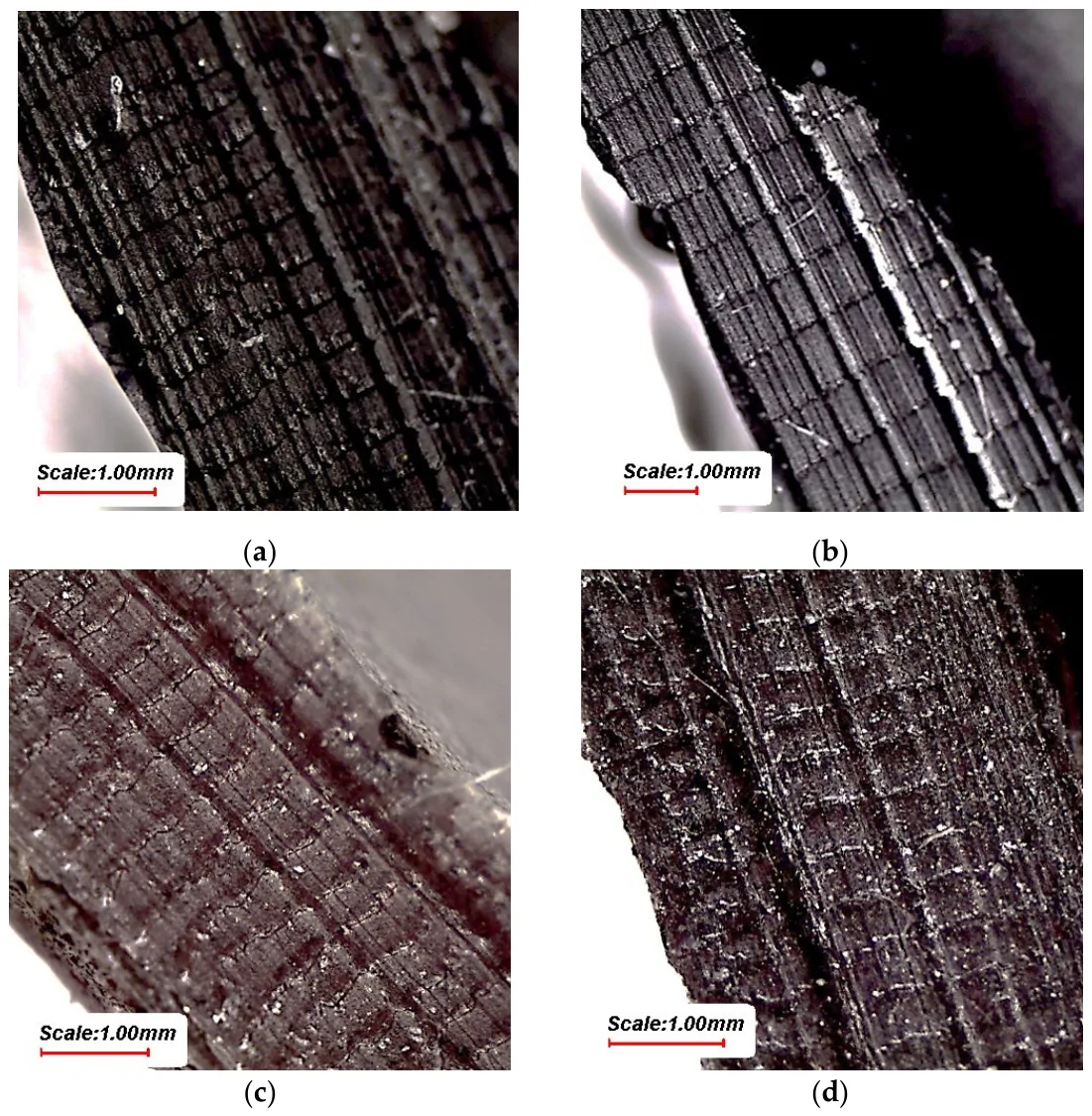

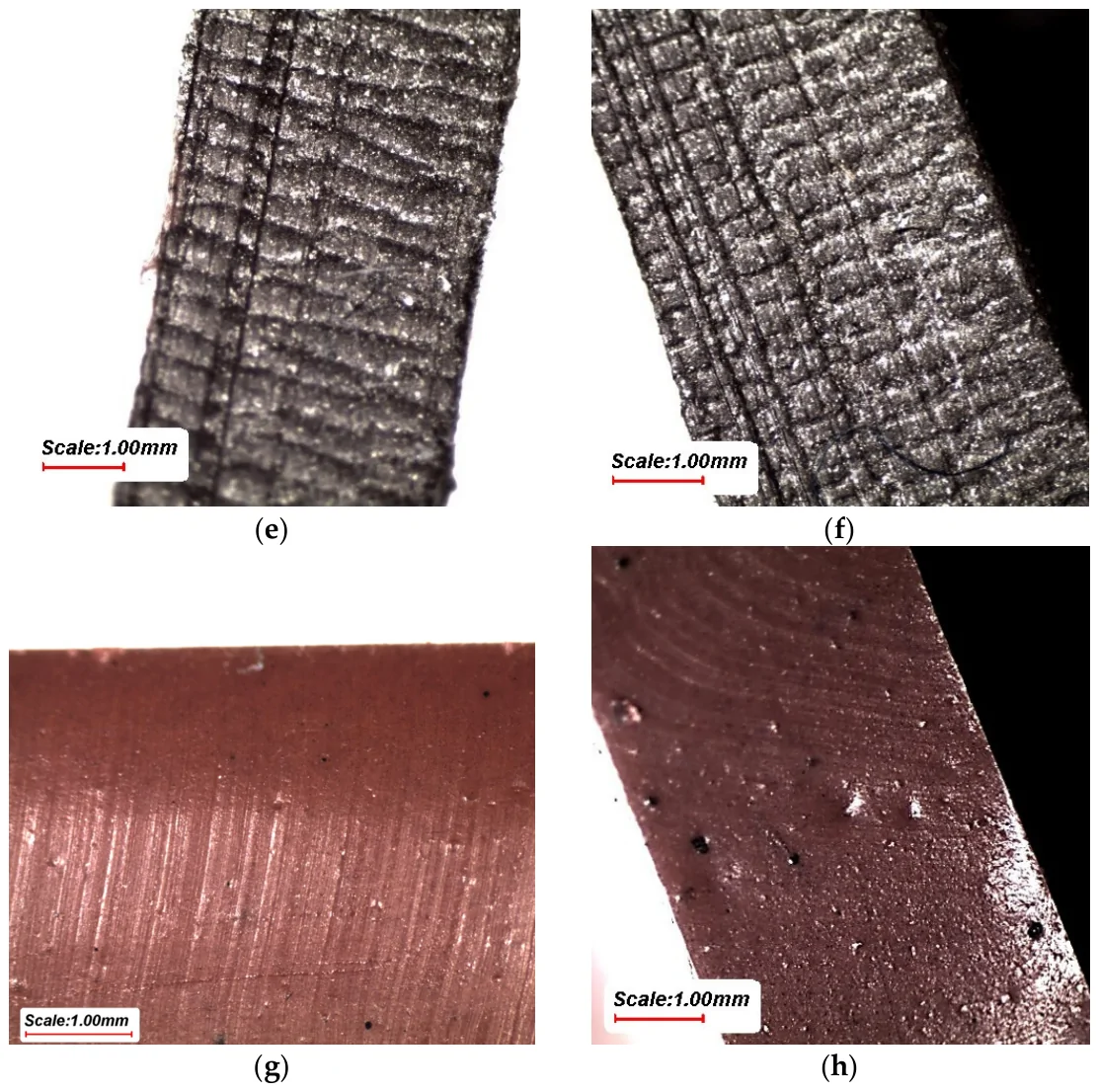
Optical micrographs composite cross-sections (**a**) S2,5; (**b**) S5; (**c**) G5; (**d**) G10; (**e**) G15; (**f**) G20; (**g**) rGO0.25; (**h**) rGO0.5.

Microscopic observations were performed on composites containing conductive carbon black, graphite flakes, and reduced graphene oxide as fillers. Figure 1 presents cross-sections of the investigated samples, providing a qualitative view of the composite cross-sections.

### 2.1. Density

The density of the materials was determined using the immersion method in accordance with PN-EN ISO 1183-1:2019-05 [36], using a Mettler-Toledo XS105 DualRange analytical balance equipped with a density determination kit (Mettler-Toledo GmbH, Schwerzenbach, Switzerland). The measurements were carried out in distilled water at a temperature of 25 ± 0.5 °C. The tests were performed on 10 specimens measuring 10 × 10 mm, taken from different locations of the cast plates.

### 2.2. Scanning Electron Microscopy (SEM)

SEM observations were performed using a ZEISS SUPRA 35 scanning electron microscope [Zeiss, Oberkochen, Germany] at an accelerating voltage of 5.00 kV, with a secondary-electron (SE) detector. Micrographs were acquired at magnifications of 250× to 500×. The composites containing conductive carbon black were analyzed in order to evaluate the composite morphology in terms of filler distribution within the matrix and the possible formation of micropores.

### 2.3. Hardness

Shore A hardness was measured in accordance with PN-EN ISO 868:2005 [37], using a Shore A durometer manufactured by Zwick & Roell (Zwick GmbH & Co., Ltd., Ulm, Germany). For each 100 × 100 mm specimen, ten hardness measurements were performed.

### 2.4. Mechanical Testing

Tensile tests were performed on five type 5A specimens in accordance with PN-EN ISO 527-2 [38]. Specimens were cut using a knife to minimize edge defects and notches that could influence the test results. The tests were carried out using a LaborTech LabTest 6.100 universal testing machine (LaborTech, Opava, Czech Republic) equipped with mechanical grips ensuring stable specimen clamping.

The tested properties, including tensile stress at break and elongation at break, were determined using a LaborTech ONE2 video extensometer (LaborTech, Opava, Czech Republic) in accordance with PN-EN ISO 527-1 [39] and PN-EN ISO 527-2. The crosshead speed was 100 mm/min. The tests were conducted in an air-conditioned laboratory at an ambient temperature of 25 ± 2 °C and a relative humidity of 50 ± 2%.

### 2.5. Surface Resistivity

Surface resistivity was measured on specimens measuring 100 × 100 × 2 mm. Prior to testing, the specimens were conditioned under laboratory conditions at a temperature of 23 ± 2 °C for 24 h. The specimen surfaces were cleaned with acetone and allowed to dry completely before measurement.

The measurements were performed using a Keithley Model 8009 resistivity test fixture connected to a Keithley 6517A electrometer (Keithley, Bracknell, UK). The surface resistivity measurement configuration was employed, utilizing a concentric electrode arrangement with a guard electrode. The specimens were placed in the test fixture, ensuring proper contact with the electrodes. Measurements were conducted at an applied voltage of 100 V with an electrification time of 15 s. Results are reported as the mean and standard deviation of three measurements.

### 2.6. Differential Scanning Calorimetry (DSC)

Differential scanning calorimetry (DSC) measurements were performed on disc-shaped specimens with a diameter of 5 mm and a mass of 12–17 mg. The analysis was carried out over a temperature range from −85 °C to 150 °C at a heating rate of 10 °C·min^−1^, followed by cooling at the same rate under a nitrogen atmosphere with a flow rate of 5 mL·min^−1^. The specimens were sealed in 40 µL aluminum crucibles. The measurements were performed using a DSC 1 instrument (Mettler-Toledo, Greifensee, Switzerland), and the data were analyzed using Mettler-Toledo STARe software 15.

## 3. Results

### 3.1. Density

**Figure 2:**
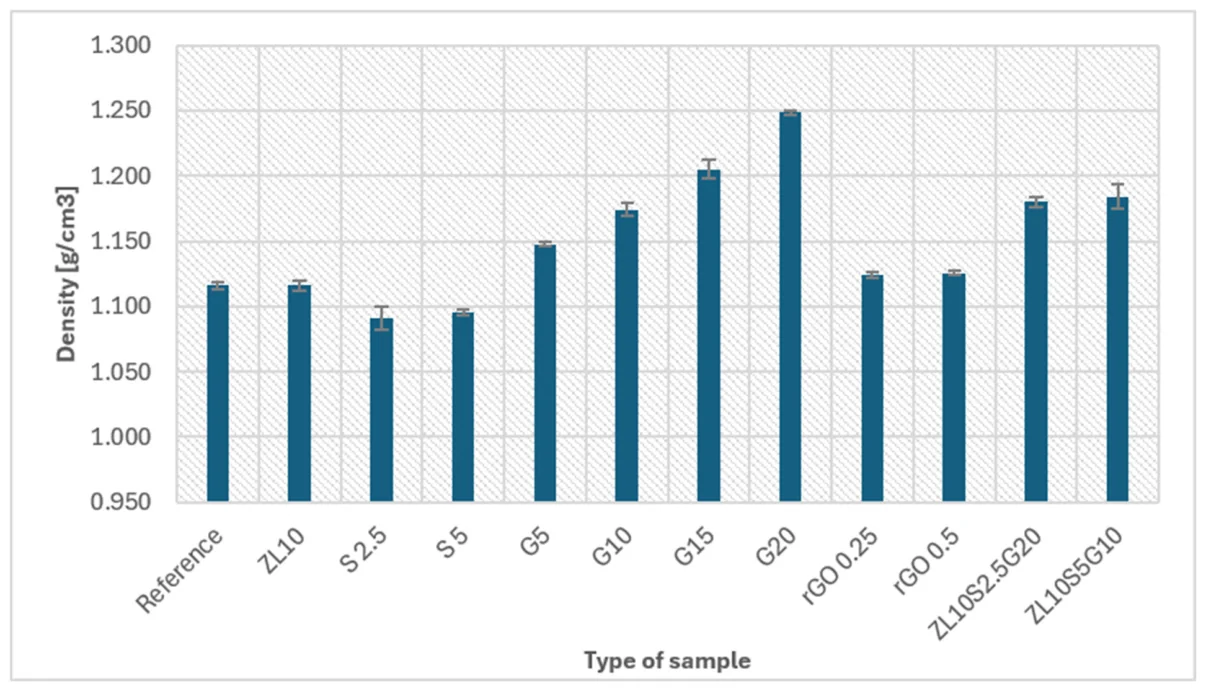
Density measurement of polyaddition PDMS AA.

Figure 2 presents the mean density values obtained for samples containing different types and contents of fillers. The presented results represent the mean values of 10 measurements, while the error bars indicate the standard deviation of the measured values.

Analysis of the density measurements of the investigated composites indicates that the density depends on the type and content of the filler used. The density of the reference sample was 1.12 g/cm^3^, and this value was used as the reference for evaluating the investigated materials and the effect of the fillers. The results showed a clear increase in density for samples filled with flake graphite. For these samples, the density increased systematically with increasing graphite content in the silicone matrix, from approximately 1.145 g/cm^3^ at a graphite content of 5 wt.% to approximately 1.25 g/cm^3^ at a graphite content of 20%. The increase in density is associated with the higher density of flake graphite, which is approximately 2.2 g/cm^3^, compared with that of the addition-cured PDMS.

**Figure 3:**
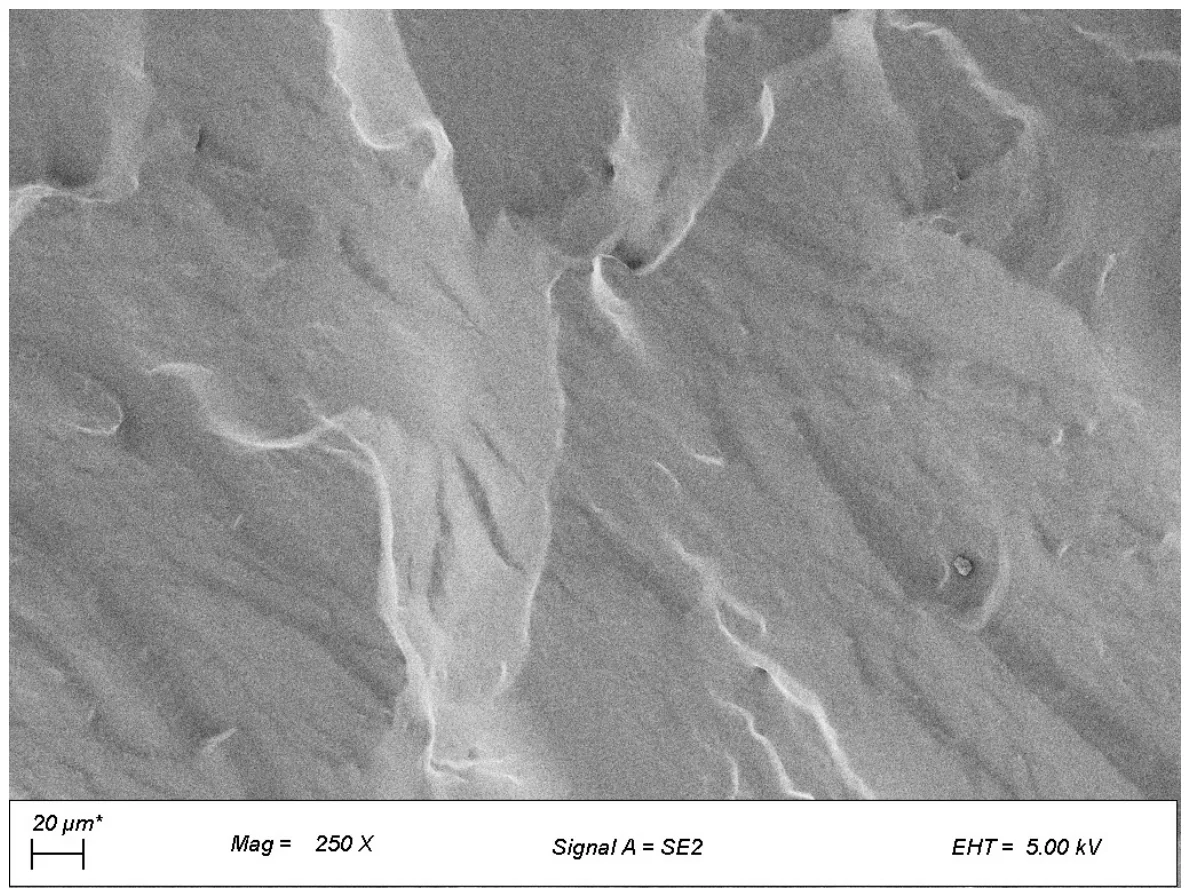
SEM of polyaddition PDMS AA—reference sample.

**Figure 4:**
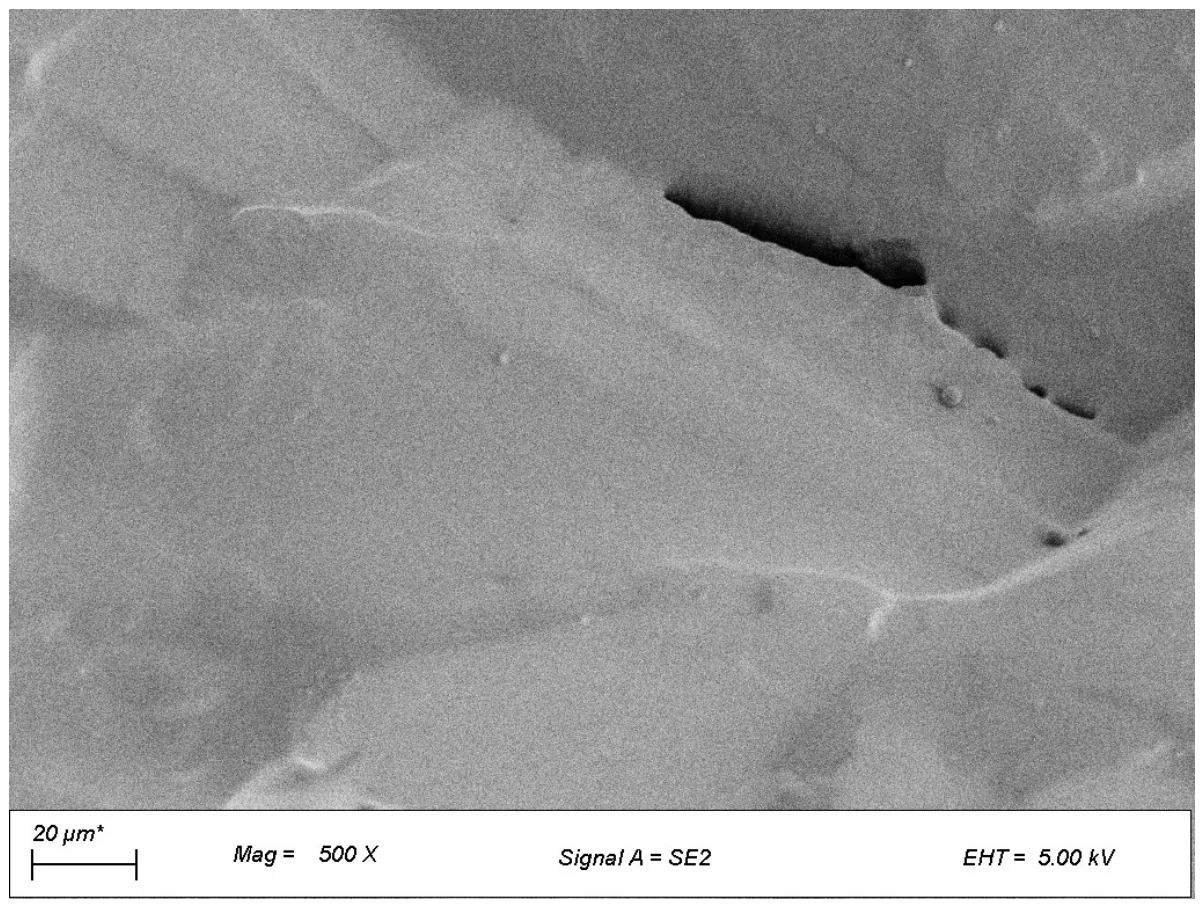
SEM of polyaddition PDMS AA—sample S2.5.

**Figure 5:**
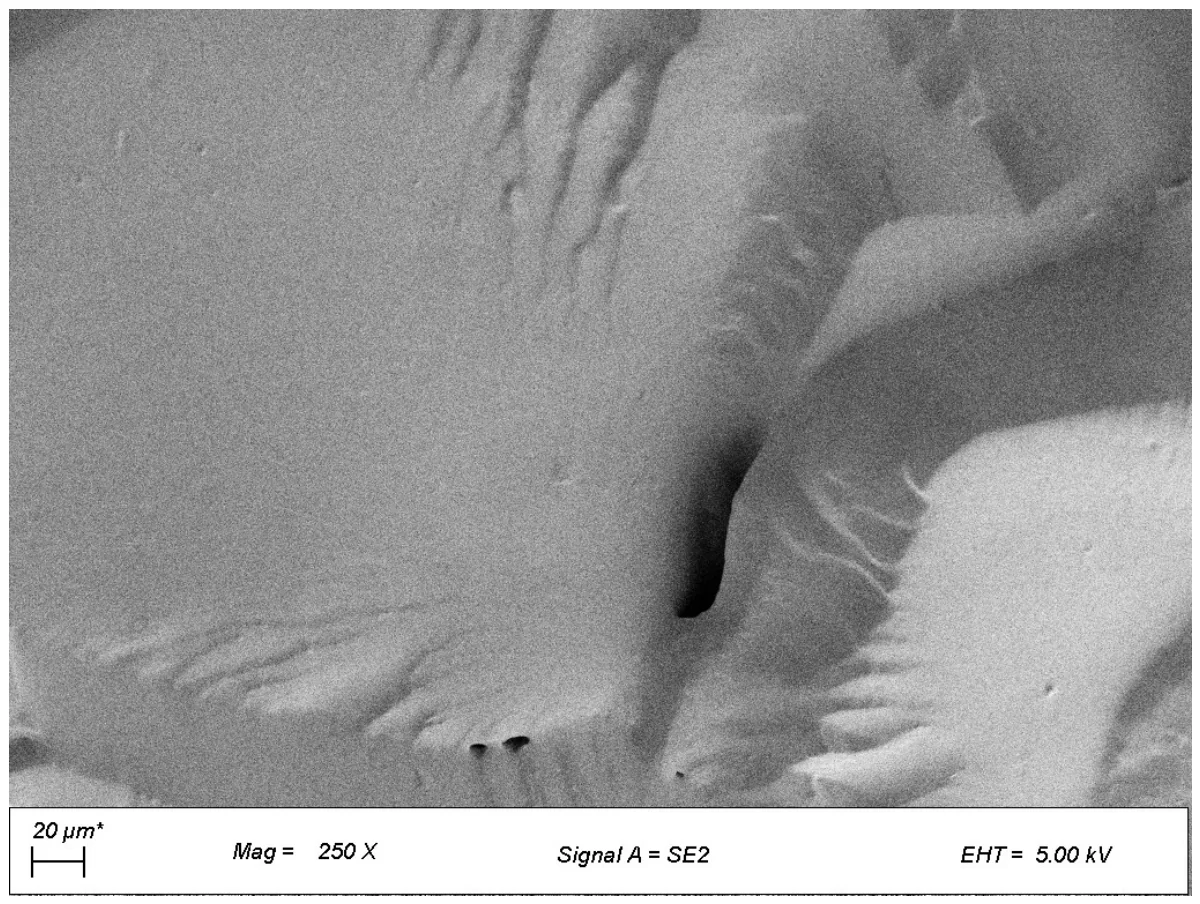
SEM of polyaddition PDMS AA—sample S5.

Samples containing 2.5 and 5 wt.% conductive carbon black showed a slight decrease in density compared with the reference sample. The decrease was approximately 2% and was most likely related to the properties of the conductive carbon black filler itself. Conductive carbon black is characterized by a high specific surface area, and even a small addition of this filler to the polymer matrix can cause a significant increase in viscosity, which makes the preparation of the mixture more difficult. The increase in viscosity observed during processing, together with more difficult degassing of the system, may promote the formation of micropores within the composite structure. Based on the obtained results, scanning electron microscopy (SEM) observations were carried out, confirming the presence of structural discontinuities in the samples (Figure 3, Figure 4 and Figure 5). The incorporation of 0.25 and 0.5% reduced graphene oxide (rGO) resulted in only a slight increase in density, which can be attributed to the relatively low amount of filler used. A similar effect was observed for the viscosity modifier (ZL), which did not affect the density compared with the reference sample.

The calculated standard deviation for each sample ranged from 0.002 to 0.011 g/cm^3^, which may indicate the absence of significant filler agglomeration in the polymer matrix and relatively uniform dispersion within the material. Based on the density measurements, it can be concluded that flake graphite had the greatest effect on this parameter.

### 3.2. Scanning Electron Microscopy (SEM)

Figure 3, Figure 4 and Figure 5 present SEM micrographs of the reference sample and composites containing conductive carbon black at filler contents of 2.5 wt.% and 5 wt.%.

SEM observations showed that the reference sample exhibited a relatively compact fracture structure, with no numerous or clearly visible voids. In the S2.5 sample, isolated local discontinuities and voids ranging from several to several tens of micrometres were observed. Local voids were also identified in the S5 sample, including a single larger elongated discontinuity; however, no clear increase in their number was observed compared with the S2.5 sample. The SEM results therefore indicate the presence of local porosity in the composites containing conductive carbon black, which is most likely associated with hindered degassing of the samples prior to casting into the moulds. The presence of such local voids may also partially explain the slight decrease in density observed for the S2.5 and S5 samples compared with the reference material, as increased structural porosity leads to a reduction in the apparent density of the composite.

### 3.3. Hardness

**Figure 6:**
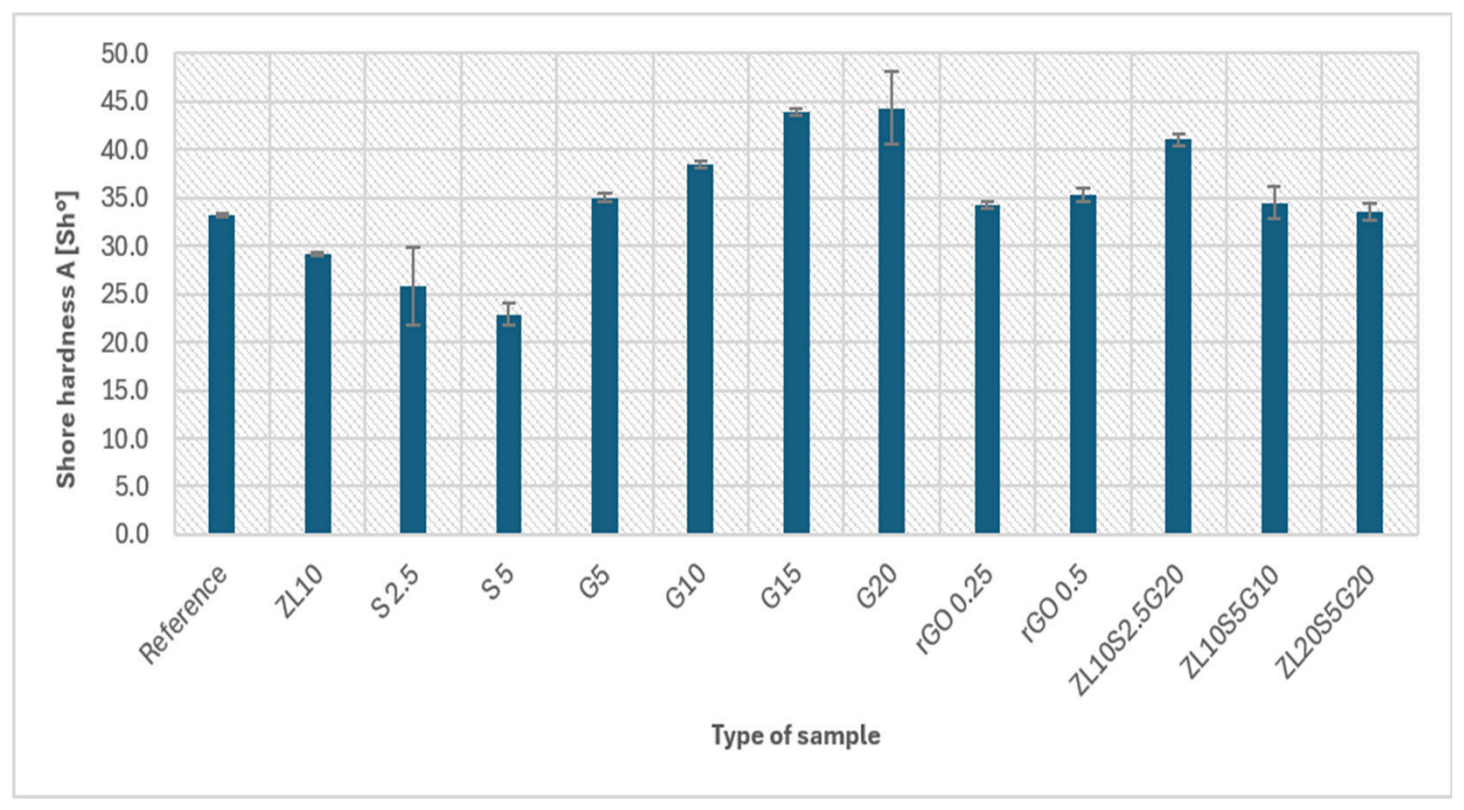
Shore hardness A of polyaddition PDMS AA.

Figure 6 presents the mean Shore A hardness values obtained for samples containing different types and contents of fillers. The presented results represent the mean values of 10 measurements, while the error bars indicate the standard deviation of the measured values.

The reference sample exhibited a Shore A hardness of approximately 33 Shore A. The other composites showed that the incorporation of fillers into the addition-cured PDMS silicone matrix significantly affected this parameter, depending on the type and amount of filler used. The most pronounced changes in hardness were observed for samples containing flake graphite. The hardness of the composites increased with increasing filler content. For sample G5, the increase was approximately 6%, for G10 approximately 15%, whereas for G15 and G20 the increases were approximately 33% and 35%, respectively, relative to the reference sample. The increase in hardness with increasing graphite content is most likely primarily related to the growing proportion of the stiffer filler phase within the flexible silicone matrix. The platelet-like morphology of graphite may restrict local deformation of the matrix and reduce the mobility of polymer chains in the interfacial region.

The opposite trend was observed for samples containing conductive carbon black. The addition of carbon black resulted in a decrease in hardness. Hardness values of approximately 25 and 23 Shore A were obtained, corresponding to decreases of approximately 23% and 30%, respectively, compared with the reference sample. The decrease in hardness may result from the increase in mixture viscosity during processing caused by the presence of carbon black, which makes degassing more difficult. As a result, structural discontinuities may form within the material, reducing its resistance to deformation. The smallest changes in hardness were observed for samples containing reduced graphene oxide (rGO), for which the increase in hardness was approximately 3–6% relative to the reference sample. Analysis of the hardness values of the multicomponent composites showed the largest increase for sample ZL10S2.5G20. This sample reached a hardness of approximately 41 Shore A, corresponding to an increase of about 24% compared with the reference sample. For samples ZL10S5G10 and ZL20S5G20, the Shore A hardness showed only a slight increase or remained comparable to that of the reference sample. Overall, the results indicate that flake graphite had the greatest influence on the increase in hardness of the investigated composites.

### 3.4. Mechanical Testing

**Figure 7:**
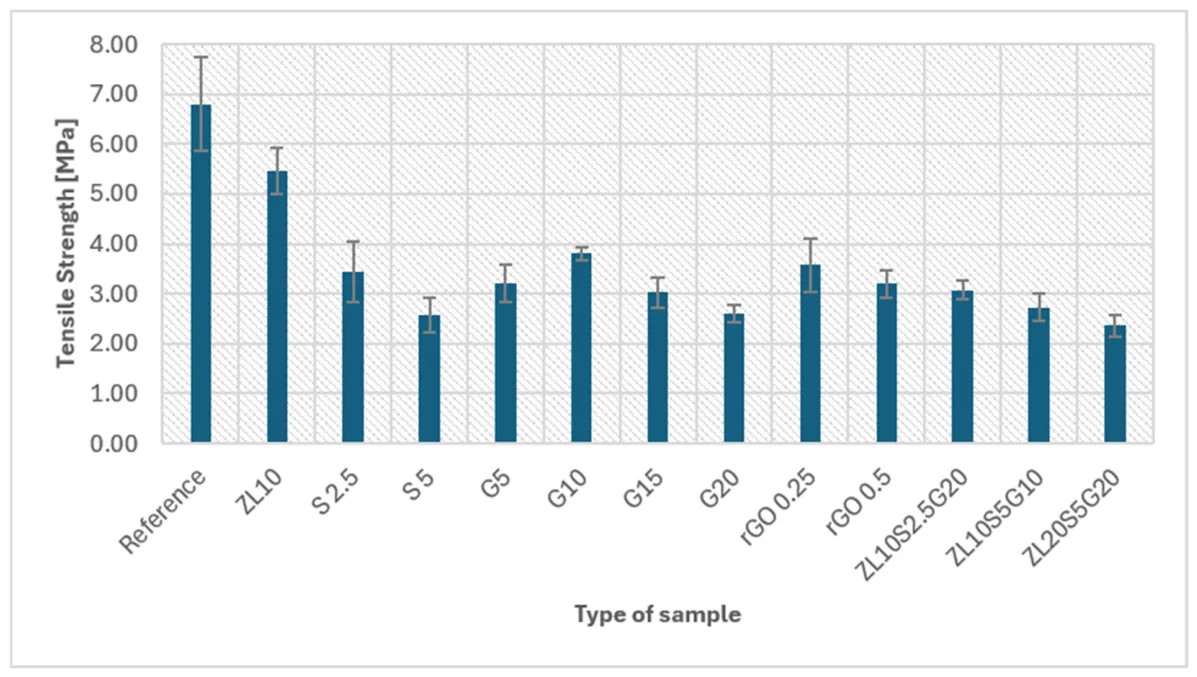
Tensile Strength of polyaddition PDMS AA.

**Figure 8:**
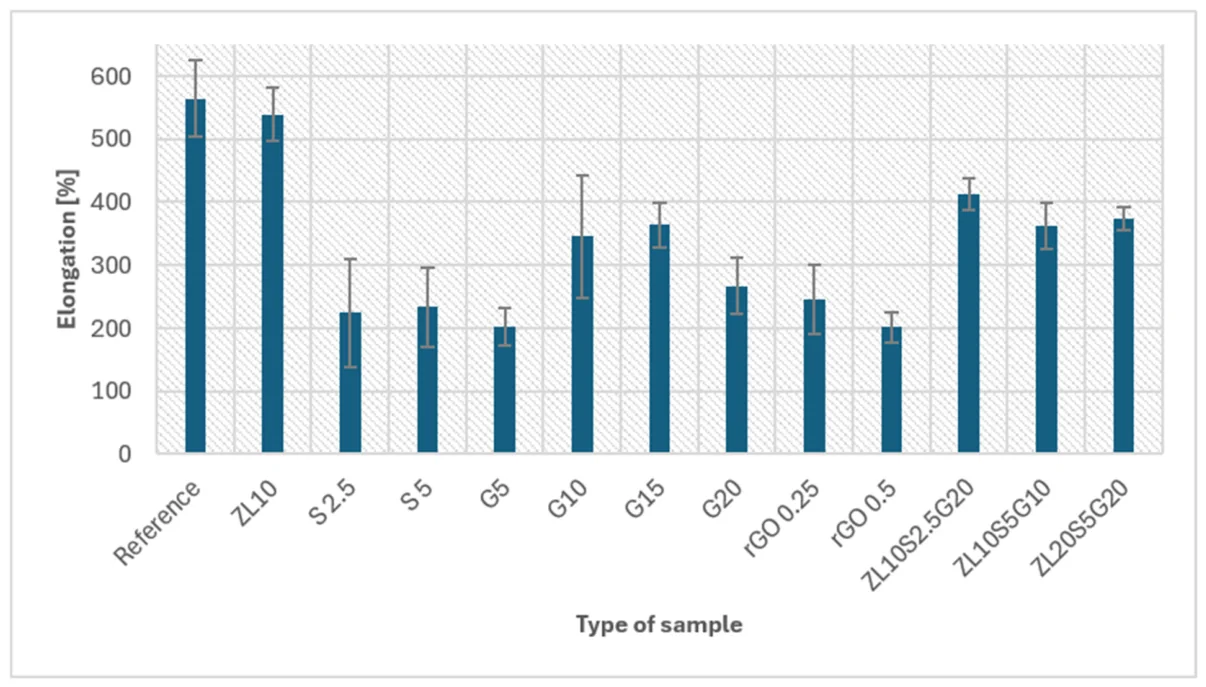
Relative Elongation of polyaddition PDMS AA.

Figure 7 and Figure 8 present the mean tensile strength and elongation at break values, respectively, obtained for samples containing different types and contents of fillers. The presented results represent the mean values of five independent measurements, while the error bars indicate the standard deviation of the measured values.

Based on the obtained results, it can be concluded that each of the introduced fillers reduced the tensile strength compared with the reference sample. The tensile strength of the reference sample was 6.8 MPa. The viscosity modifier had the smallest effect on tensile strength, reducing its value by approximately 20% relative to the reference sample.

The greatest decrease in tensile strength was observed for the samples filled with conductive carbon black. At a carbon black content of 2.5 wt.%, the tensile strength decreased to 3.4 MPa, whereas at 5 wt.% carbon black it decreased further to 2.5 MPa. As the carbon black content increases, the ability of the PDMS matrix to undergo large deformations becomes limited. In addition, the sharp decrease in surface resistivity at a carbon black content of 5 wt.% indicates the formation of an extensive filler network, which may disrupt the continuity of the polymer phase. The structural discontinuities observed in the SEM images may further facilitate damage initiation during tensile deformation.

For the samples containing flake graphite, sample G5 exhibited a tensile strength of 3.0 MPa, which increased to 3.8 MPa when the graphite content was increased to 10%. For the subsequent samples containing 15 wt.% and 20 wt.% graphite, the tensile strength again showed a decreasing trend. Increasing the graphite content from 5 wt.% to 10% may have promoted more effective stress transfer between the matrix and the stiffer filler phase. At higher graphite contents, however, the increasing number of contacts between graphite platelets, together with the reduced continuity and deformability of the polymer phase, may lead to stress localization and, consequently, to a renewed decrease in tensile strength.

The incorporation of rGO also resulted in a decrease in tensile strength compared with the reference sample. Values of approximately 3.6 MPa for rGO0.25 and 3.2 MPa for rGO0.5 were obtained. The multicomponent samples exhibited tensile strength values in the range of approximately 2.3–3.1 MPa.

Elongation at break, like tensile strength, decreased compared with the reference sample, for which the elongation at break reached approximately 560%. The viscosity modifier had the smallest effect on elongation, causing only a slight change in this parameter, with the sample reaching a value of approximately 540%. As in the case of tensile strength, the greatest reduction in elongation at break was observed for the samples containing conductive carbon black. Samples S2.5 and S5 exhibited elongation values of approximately 220% and 230%, respectively, corresponding to a reduction of more than half compared with unmodified PDMS. For the sample containing 5 wt.% flake graphite, an elongation at break of approximately 190% was obtained. This value increased to approximately 345–360% when the graphite content was increased by a further 5–10%. However, a further increase in filler content to 20% resulted in a decrease in elongation at break to approximately 265%.

The addition of rGO also reduced the elongation at break. A value of approximately 240% was obtained for rGO0.25, whereas increasing the rGO content to 0.5 wt.% led to a further decrease to approximately 195%. Due to the high specific surface area and high aspect ratio of rGO, even a small amount of this filler may significantly restrict the mobility of PDMS macromolecules. The multicomponent composite samples exhibited higher elongation at break than most samples containing single fillers. The highest value, approximately 410%, was obtained for ZL10S2.5G20, whereas samples ZL10S5G10 and ZL20S5G20 reached approximately 360–370%. These results suggest that selected combinations of carbon black, graphite, and the viscosity modifier may partly mitigate the loss of flexibility; however, filler dispersion was not quantified.

### 3.5. Surface Resistivity

**Figure 9:**
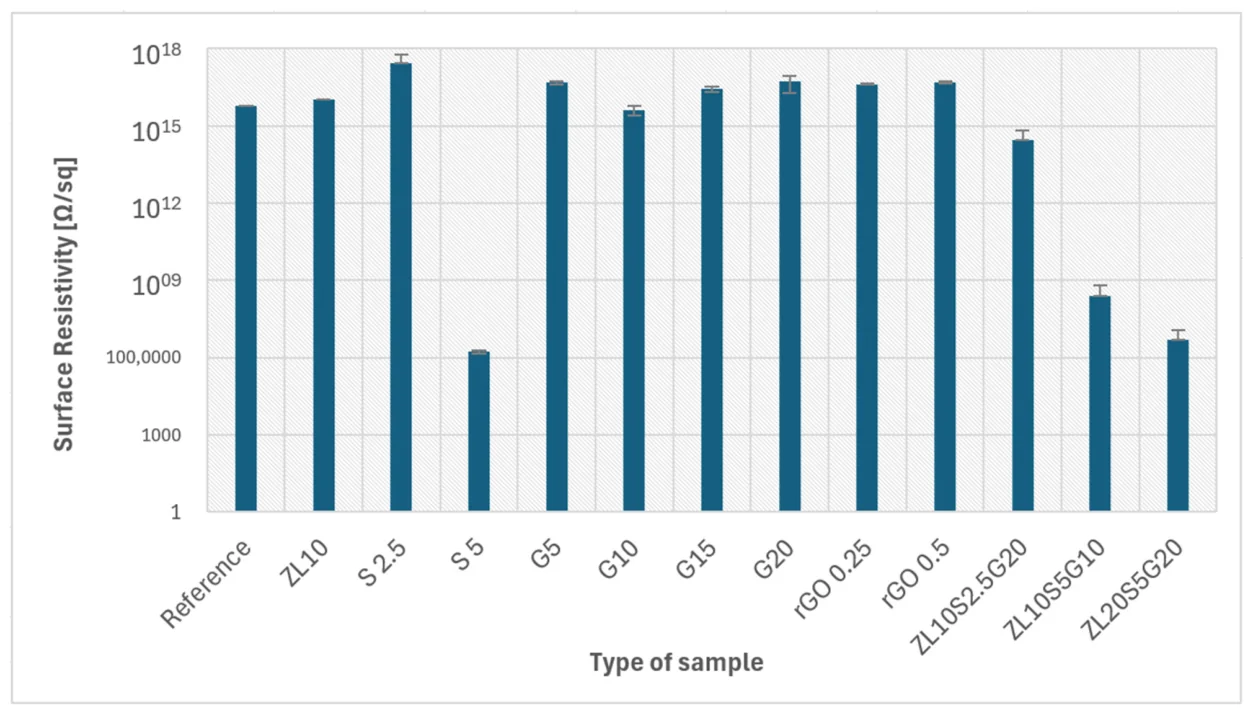
Surface resistivity of polyaddition PDMS AA.

Figure 9 presents the mean surface resistivity values obtained for samples containing different types and contents of fillers. The presented results represent the mean values of three independent measurements, while the error bars indicate the standard deviation of the measured values.

Analysis of the surface resistivity measurements indicates that the electrical properties of the composites depend on the type and content of filler incorporated into the PDMS matrix. The reference sample exhibited a high surface resistivity of approximately 10^16^ Ω/sq, which is characteristic of an electrically insulating material. Most of the investigated samples, including those containing the viscosity modifier, flake graphite, and rGO, did not show a decrease in surface resistivity. This indicates that these materials retained their insulating properties and that no effective electrically conductive network was formed.

The most pronounced effect on electrical properties was observed for conductive carbon black. At a filler content of 2.5 wt.%, the composite retained its insulating properties, whereas increasing the carbon black content to 5 wt.% resulted in a sharp decrease in the mean surface resistivity to approximately 10^6^ Ω/sq. This pronounced change suggests the possible formation of an electrically conductive network within the PDMS matrix, which may facilitate electrical charge transport. Based on these observations, the electrical percolation threshold is likely to lie between 2.5 and 5 wt.% conductive carbon black; however, its exact value cannot be determined from the investigated filler concentrations alone.

In the case of the multicomponent systems, the carbon black content played a key role. Sample ZL10S2.5G20 exhibited a surface resistivity of approximately 10^14^ Ω/sq, whereas increasing the carbon black content to 5 wt.% in samples ZL10S5G10 and ZL20S5G20 reduced the surface resistivity to approximately 10^8^ and 10^7^ Ω/sq, respectively.

### 3.6. Differential Scanning Calorimetry (DSC)

**Figure 10:**
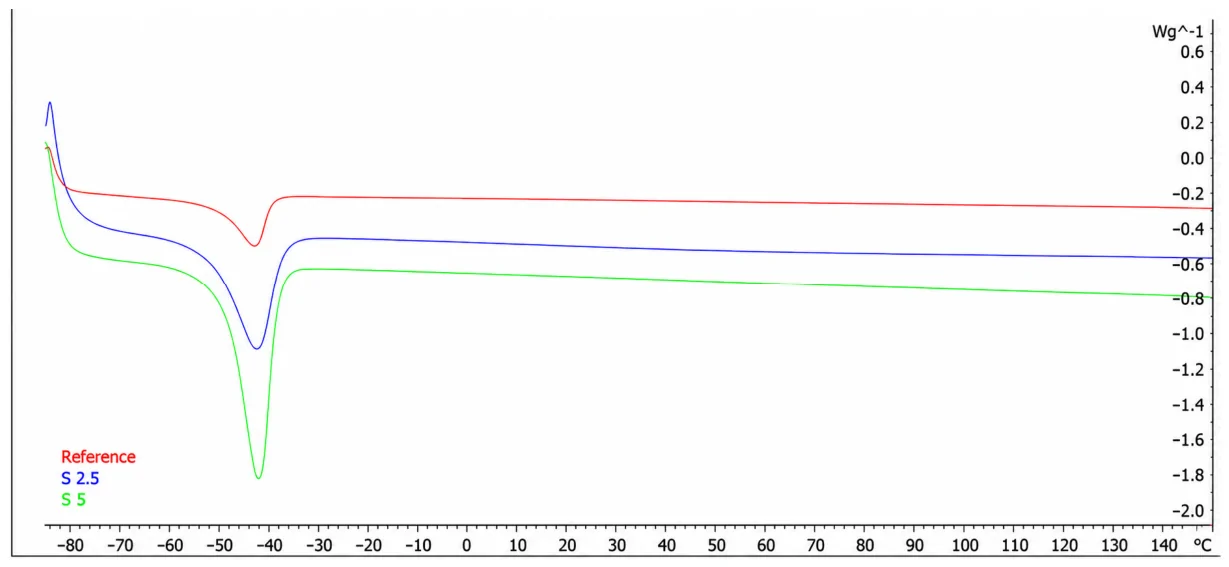
DSC of polyaddition PDMS AA with conductive carbon black.

**Figure 11:**
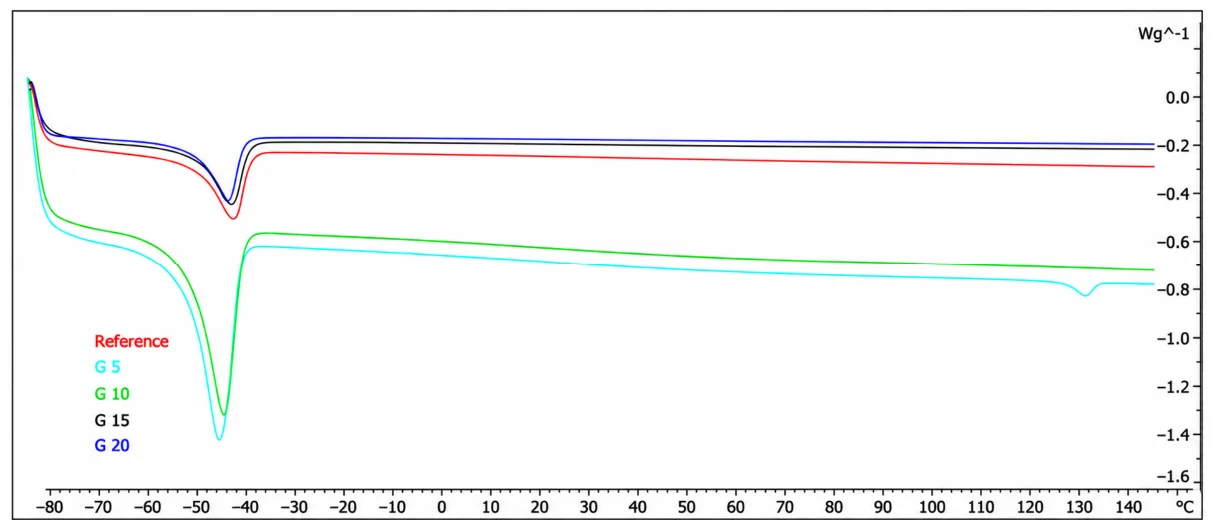
DSC of polyaddition PDMS AA with flake graphite.

Figure 10 and Figure 11 present the DSC curves obtained for the reference sample and the composites containing carbon fillers in the form of conductive carbon black and flake graphite. The presented thermograms and thermal analysis results correspond to the first heating cycle, allowing the evaluation of thermal transitions occurring in the materials while taking into account their initial thermal history resulting from specimen preparation and storage.

The characteristic thermal parameters determined from the DSC curves for the reference sample and the composites containing conductive carbon black are summarized in Table 3. The peak temperature remained essentially unchanged, ranging from −42.79 °C for the reference sample to −42.13 °C for the S5 sample. Similarly, the onset temperature did not show a clear dependence on carbon black content and ranged from −51.89 to −48.98 °C. These observations suggest that conductive carbon black had little effect on the characteristic temperature of the observed thermal transition in the PDMS matrix.

A much more pronounced effect of carbon black content was observed for the transition enthalpy. For the reference sample, the ΔH value was −14.46 J/g, whereas after the addition of 2.5 wt.% carbon black, its absolute value increased to 40.15 J/g, and for the sample containing 5 wt.% carbon black it reached 56.69 J/g. These results indicate that the presence of carbon black primarily affects the intensity of the observed transition rather than its temperature. The increase in the absolute value of the enthalpy with increasing filler content may indicate changes in the degree of ordering of the polymer phase and an increase in the fraction of structures participating in the observed transition.

The characteristic thermal parameters determined from the DSC curves for the reference sample and the composites containing flake graphite are summarized in Table 4. The incorporation of graphite affected both the characteristic transition temperature and the transition enthalpy; however, the observed changes were non-monotonic. For the G5 and G10 samples, the peak temperature shifted toward lower values, reaching −45.62 °C and −44.60 °C, respectively, compared with −42.79 °C for the reference sample. At the same time, the absolute value of the transition enthalpy increased from 13.81 J/g for the reference sample to 37.69 J/g for G5 and 35.76 J/g for G10.

With a further increase in graphite content, a pronounced decrease in the absolute value of the enthalpy was observed. For the G15 and G20 samples, the values were 12.54 and 11.54 J/g, respectively, which were close to that obtained for the reference material. These results indicate that the effect of flake graphite depends on its concentration. At lower filler contents, graphite may promote the formation of more ordered polymer domains. At higher filler contents, this effect may be limited by reduced polymer-chain mobility, increased interfacial interactions, and a reduction in the space available for macromolecular reorganization.

## 4. Conclusions

The conducted studies demonstrated that the type, morphology, and content of carbon fillers have a significant influence on the electrical and physicomechanical properties of addition-cured PDMS-based composites. Among the investigated fillers, conductive carbon black was the most effective in reducing surface resistivity. The composite containing 2.5 wt.% carbon black retained its insulating properties, whereas increasing the filler content to 5 wt.% reduced the mean surface resistivity to approximately 10^6^ Ω/sq, compared with approximately 10^16^ Ω/sq for the unfilled PDMS. Such a sharp decrease may indicate the onset of the formation of continuous an electrically conductive network. The electrical percolation threshold for the investigated PDMS–conductive carbon black system is likely to lie between 2.5 and 5 wt.% filler content.

The high effectiveness of conductive carbon black may be related to its morphology and the ability of its small particles to form branched aggregates. As the filler content increases, the average distance between conductive regions decreases and the probability of direct contact between them increases, which, after exceeding a critical concentration, leads to the formation of a three-dimensional conductive network. In contrast to carbon black, flake graphite at contents of up to 20 wt.% and rGO at contents of up to 0.5 wt.% did not result in the formation of effective electrically conductive network. In the case of graphite, this may be associated with its larger particle size, platelet morphology, and the dependence of conductivity on platelet orientation and interparticle contact. For rGO, the applied concentration may have been insufficient to form a continuous conductive network.

The improvement in electrical properties was accompanied by deterioration of some mechanical properties. The tensile strength of the unmodified PDMS was approximately 6.8 MPa, whereas the addition of 2.5 and 5 wt.% conductive carbon black reduced it to approximately 3.4 and 2.5 MPa, respectively. A similar trend was observed for elongation at break, which decreased from approximately 560% for the reference sample to approximately 220–230% for the composites containing carbon black. As the carbon black content increases, the ability of the PDMS matrix to undergo large deformations becomes increasingly limited. At the same time, the sharp decrease in surface resistivity at a carbon black content of 5% indicates the formation of an extensive carbon black network, which may reduce the effective continuity of the polymer phase. Structural discontinuities observed in the SEM images may additionally promote damage initiation during tensile deformation. This mechanism is consistent with the simultaneous reduction in both tensile strength and the ability of the material to undergo large deformations.

Flake graphite, in contrast, caused a systematic increase in Shore A hardness with increasing filler content. For the G15 and G20 samples, the increase was approximately 33–35% compared with the reference material. This effect can be attributed to the increasing proportion of the graphite phase. Increasing the graphite content from 5 wt.% to 10 wt.% resulted in an increase in tensile strength from approximately 3.0 to 3.8 MPa, which may indicate more effective stress transfer between the matrix and the stiffer filler phase. With a further increase in graphite content, a decrease in tensile strength was again observed. In this case, the increasing number of contacts between graphite platelets, together with the reduced continuity and deformability of the polymer phase, may promote stress localization and earlier damage initiation.

Significant results were obtained for the multicomponent formulations. Composites containing 5 wt.% conductive carbon black in combination with flake graphite and a viscosity modifier exhibited surface resistivity values of approximately 10^7^–10^8^ Ω/sq, while maintaining elongation at break at approximately 360–370%. These values were considerably higher than those obtained for materials containing conductive carbon black alone. The ZL10S2.5G20 sample exhibited an elongation at break of approximately 410%; however, its surface resistivity remained relatively high, at approximately 10^14^ Ω/sq. These results confirm the dominant role of conductive carbon black in the formation of a charge-transport electrically conductive network, whereas the presence of graphite and the viscosity modifier may favorably affect the distribution of the carbon phase and reduce the loss of flexibility. In hybrid systems, larger graphite platelets may form elements of the conductive framework, while smaller carbon black aggregates may locally occupy the spaces between them and create additional electrical connections. This concept constituted one of the assumptions underlying the design of the investigated formulations.

SEM observations revealed the presence of local voids in composites containing conductive carbon black. Their formation may be associated with hindered degassing of the mixtures prior to casting, particularly due to increased system viscosity and the high specific surface area of carbon black. Residual entrapped air may, in turn, partially explain the slight decrease in the density of carbon-black-containing composites compared with the reference sample. It should be noted, however, that SEM observations are local in nature and do not allow quantitative determination of the overall porosity of the material.

DSC analysis showed that conductive carbon black had a relatively small effect on the characteristic temperature of the observed thermal transition, while causing a pronounced increase in the absolute value of the enthalpy. This behavior may indicate that the filler primarily affects the degree of ordering and the fraction of the matrix participating in the transition, while having a smaller effect on the transition temperature itself. In the case of flake graphite, the relationship was non-monotonic. The highest absolute enthalpy values were obtained for the G5 and G10 samples, whereas for G15 and G20 the values approached that of the reference sample. This may indicate the occurrence of two competing effects. At lower filler contents, the graphite surface may promote local ordering of PDMS chains, whereas at higher filler contents, restricted macromolecular mobility and an increased proportion of interfacial regions may hinder further reorganization of the polymer chains.

Overall, conductive carbon black was the most effective filler in reducing the surface resistivity of PDMS, although the formation of a continuous conductive network was accompanied by deterioration of some mechanical properties. The results also suggest that selected combinations of fillers with different morphologies may partially mitigate these mechanical losses. Hybrid filler systems may therefore offer a route to flexible silicone composites combining lower surface resistivity with substantial deformability.

## Figures and Tables

**Table 1:** Material properties based on manufacturer data [34].

Properties	Standards	Unit	AA
Mixing proportion (base:catalyst)	-		100:10
Density 25 °C	ISO 1183-1 [35]	g/cm^3^	1.12
Brookfield viscosity	-	mPa s	20,000
Lifetime 25 °C	-	min	60
Demoulding time 23 °C	-	h	16

**Table 2:** Designations of the tested PDMS AA-based samples.

Designation	Type of Filler	Amount of Filler [%]
Reference sample	none	none
ZL10	Viscosity modifier	10
S2.5	Conductive carbon black	2.5
S5	Conductive carbon black	5
G5	Flake graphite	5
G10	Flake graphite	10
G15	Flake graphite	15
G20	Flake graphite	20
rGO0.25	Reduced graphene oxide	0.25
rGO0.5	Reduced graphene oxide	0.5
ZL10S2.5G20	Viscosity modifier Conductive carbon black Flake graphite	10 2.5 20
ZL10S5G10	Viscosity modifier Conductive carbon black Flake graphite	10 5 10
ZL20S5G20	Viscosity modifier Conductive carbon black Flake graphite	20 5 20

**Table 3:** Characteristic thermal parameters determined from the DSC curves for samples containing conductive carbon black.

Type of Sample	T Onset [°C]	T Peak [°C]	ΔH [J/g]
Reference	−50.80	−42.79	−14.46
S 2.5	−51.89	−42.42	−40.15
S 5	−48.98	−42.13	−56.69

**Table 4:** Characteristic thermal parameters determined from the DSC curves for samples containing flake graphite.

Type of Sample	T Onset [°C]	T Peak [°C]	ΔH [J/g]
Reference	−50.70	−42.79	−13.81
G 5	−52.43	−45.62	−37.69
G 10	−52.16	−44.60	−35.76
G 15	−50.60	−43.25	−12.54
G 20	−50.84	−43.93	−11.54

## Data Availability

The original contributions presented in this study are included in the article. Further inquiries can be directed to the corresponding author.
